# Cryoablation of Anteroseptal Accessory Pathways with a His Bundle Electrogram on the Ablation Catheter

**DOI:** 10.1016/s0972-6292(16)30816-6

**Published:** 2014-12-05

**Authors:** Leonardo Liberman, David S Spar, Mary C Nash, Eric S Silver

**Affiliations:** Pediatric Arrhythmia Service, Department of Pediatrics, Morgan Stanley Children Hospital, Columbia University, New York, New York

**Keywords:** Cryoablation, Anteroseptal Accessory Pathways

## Abstract

**Background:**

Radiofrequency catheter ablations of anteroseptal (AS) accessory pathways (AP) in pediatric patients have higher incidence of atrioventricular (AV) block than other AP locations. We report our experience using cryoablation in pediatric patients where a His bundle electrogram was noted on the ablation catheter at the site of the successful ablation.

**Methods and Results:**

We retrospectively reviewed all patients ≤21 years that underwent cryoablation for an AS AP from 2005 to 2012 at our institution (n=70). Patients with a His bundle electrogram noted on the cryoablation catheter at the location of the successful lesion were identified (n=6, 8.5%). All six patients had ventricular preexcitation. Median age of 15.9 years (7.2 - 18.2). AV nodal function was monitored during the cryoablation with intermittent rapid atrial pacing conducted through the AV node (n=2), with atrial extra-stimulus testing (n=2), or during orthodromic reentrant tachycardia (n=2). Acute success occurred in all patients. Two patients had early recurrence of AP conduction. Both patients underwent a second successful cryoablation, again with a His bundle electrogram on the cryoablation catheter. At a median follow-up of 13 months (3 to 37 months) there was no recurrence of accessory pathway conduction and AVN function was normal.

**Conclusion:**

In a small number of pediatric patients with AS AP with a His bundle electrogram seen on the ablation catheter, the use of cryotherapy was safe and effective for elimination of AP conduction without impairment of AV nodal conduction.

## Introduction

Electrophysiologic testing with catheter ablation of accessory pathways is indicated in some patients with Wolf-Parkinson-White syndrome or as an elective procedure offered as an alternative to chronic antiarrhythmic drug therapy [1]. Ablation of accessory pathways located close to the conduction system are often challenging. Prior to the introduction of cryotherapy, ablation of anteroseptal pathways with radiofrequency energy had a lower success rate than other locations with high incidence of AV block [2,3]. Although the use of cryoenergy had not significantly changed the acute success and recurrence rates after ablation of anteroseptal accessory pathways, it has certainly decreased the incidence of heart block [4]. It is possible that the success rate considering intention to treat anteroseptal accessory pathways has increased with the use of cryoenergy, however this has not been proven. A His bundle electrogram at the ablation site has been used as a predictor for the development of AV block in patients undergoing AV nodal ablation [5]. The presence of a His bundle electrogram during ablation of parahisian accessory pathways presents a challenge. Ablation using radiofrequency energy would not be delivered in such location in an elective ablation of an anteroseptal accessory pathway. Cryotherapy offers a safer alternative, since permanent heart block has not been described using this technology.We describe our experience using cryotherapy for the ablation of parahisian accessory pathways with a His bundle electrogram seen on the ablation catheter at site of the successful ablation location and describe the techniques used to monitor atrioventricular nodal function during the ablation.

## Methods

### Study Design

After Institutional Review Board approval, we performed a retrospective review of patients that underwent an electrophysiology study with ablation of an anteroseptal accessory pathway. Patients less than or equal to age 21 years who underwent a catheter ablation between 2005 and 2012 were included. Patients who underwent catheter ablation with a cryoablation catheter with a His bundle electrogram noted on the cryoablation catheter at the location of the successful lesion were further analyzed.

### Electrophysiology Testing

General anesthesia was used for all electrophysiology studies. Twelve-lead surface electrocardiograms and bipolar intracardiac electrograms filtered at 30 to 500 Hz were recorded at a rate of 200 mm/sec using a EP Workmate System (St Jude, West Berlin, NJ). Pacing catheters were inserted via the right and left femoral veins and through the right internal jugular vein. The catheters were positioned in the right atrial appendage, right ventricular apex, the His bundle area and within the coronary sinus. Baseline intracardiac intervals, incremental atrial pacing, atrial extra-stimulus testing, incremental ventricular pacing and ventricular extra-stimulus testing were performed at baseline conditions and with an infusion of isoproterenol (0.01 to 0.02 μg/kg/min) if needed to induce tachycardia.

### Ablation technique

**Figure 1:**
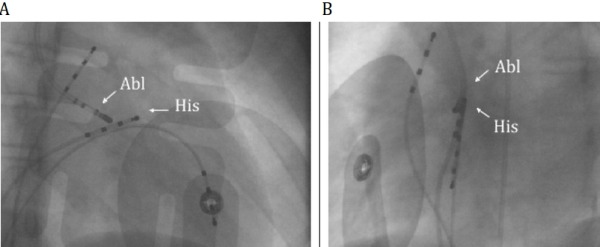
Right anterior oblique (A) and left anterior oblique (B) views demonstrating proximity of the His catheter (His) and the ablation catheter (Abl).

**Figure 2:**
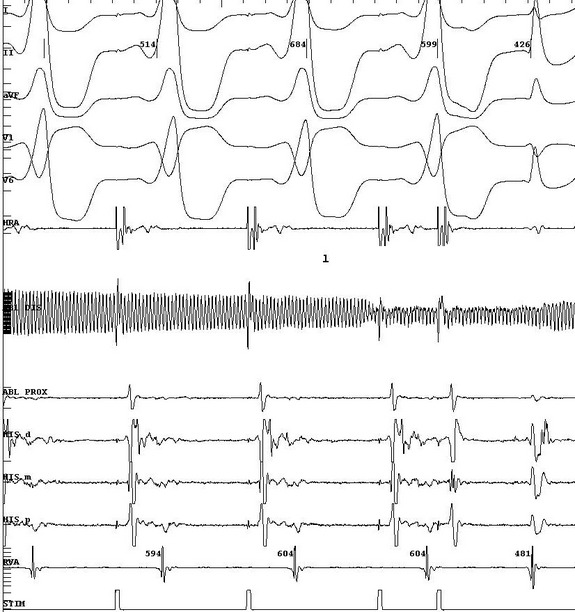
Pacing maneuver during a cryo-application. Atrial extrastimulous testing at a drive train of 600 msec with an extrastimulus at 270 msec demonstrating conduction via the AV node. ECG leads I, II, aVF, V1, V6. HRA = high right atrium, AblD = distal ablation catheter, AblP = proximal ablation catheter, His = his catheter (D= distal, M=medial, P=proximal), RVA= right ventricular apex, Stim = stimulation channel.

**Figure 3:**
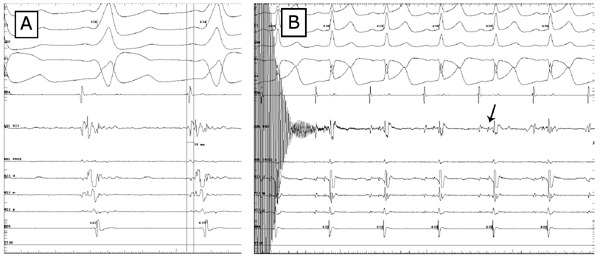
Panel A: signal at the site of success with a local AV time of 38 msec. The ventricular electrogram in the AblD is 55 msec prior to the initiation of the delta wave. Panel B: His electrogram seen on the ablation catheter at the end of the cryoapplication (arrow).

Mapping of the accessory pathway was performed using standard techniques [6]. Once the accessory pathway location was identified to be in the anteroseptal area the decision was made to use a cryocatheter (Medtronic, Inc. Minneapolis, MN) for the ablation. A 4 or 6 mm tip catheter was used and mapping of the anteroseptal area was performed from the right femoral vein or the right internal jugular vein (Figure 1). Once a target site was identified based on either a continuous atrioventricular signal in sinus or atrial paced rhythm or closely coupled local ventriculoatrial signal during orthodromic reentry tachycardia, pacing maneuvers were performed to determine the presence of a His bundle electrogram on the ablation catheter. The following pacing maneuvers were used: 1) rapid atrial pacing at a cycle length with conduction only via the AV node, 2) atrial extra-stimulus testing with a drive train of 4 beats and a premature beat with a coupling interval shorter than the accessory pathway effective refractory period if conduction only via the AV node was seen at that coupling interval (Figure 2) or 3) induction of orthodromic reentry tachycardia. If a His bundle electrogram was seen in the chosen location, ablation was delivered and the pacing maneuvers described above were repeated during the cryo-application to monitor AV nodal function. If accessory pathway conduction was not interrupted within 30 sec of freezing, the lesion was halted and the catheter repositioned. Once accessory pathway interruption was obtained a full 4 minute lesion was placed and at least two more insurance lesions with a freeze-thaw-freeze technique were placed [7]. A His electrogram was seen in all the patients at the successful ablation location after the conduction of the accessory pathway was eliminated (Figure 3). Post ablation testing was performed for 30 minutes. Acute success was defined as no accessory pathway conduction at the end of the study. Recurrence of accessory pathway conduction at follow-up was defined as return of documented tachycardia or ventricular preexcitation on surface electrocardiogram.

## Results

### Demographic and EP study characteristics

There were 70 patients with an anteroseptal accessory pathway that underwent catheter ablation during the study period; six patients (8.5%) were identified as having had a His bundle electrogram on the ablation catheter at the site of success, all with ventricular preexcitation. Four patients were male,the median age at the time of the ablation was 15.9 years (range: 7.2 - 18.2) and the median weight was 68 kg (range: 23 - 82). At baseline, the mean PR interval measured 94 ± 8 msec, the AH interval was 69 ± 17 msec and the HV interval measured -25 ± 25 msec. The accessory pathway effective refractory period was 350 ± 80 msec. Orthodromic reentry tachycardia was induced in 5 patients with a mean tachycardia cycle length of 348 ± 29 msec.

### Ablation characteristics

Acute success was achieved in all patients (100% acute success). The catheter approach during the successful lesion was via the right femoral vein in four patients and the right internal jugular vein in two. A long sheath was not used when approaching from the right femoral vein. Two patients were ablated with a 4 mm tip cryocatheter and four patients with a 6 mm cryocatheter. The median time from reaching a temperature of -70 Celsius to loss of accessory pathway conduction was 9.0 sec (range 1.3 to 14 seconds). AV nodal function was monitored during the cryoablation with rapid atrial pacing conducted only through the AV node (n=2), atrial extra-stimulus testing (n=2), or during orthodromic reentrant tachycardia (n=2). One patient had transient complete AV block and another patient had AH prolongation during cryoablation, both resolved immediately after discontinuation of the refrigerant application. The catheter was repositioned in one patient and moved from the right internal jugular vein to the right femoral vein in the second. Again a His bundle was noted on the distal ablation catheter and further lesions were delivered with elimination of accessory pathway conduction without heart block or AH prolongation in both patients.

Post ablation testing showed an average AH interval of 71 ± 20 msec (not significantly different from prior to the ablation), HV time of 50 ± 7 msec (vs. -25 ± 25 prior to ablation, p<0.01) and Wenckebach cycle length of 327 ± 37 msec. AV nodal function at the end of the case was normal in all patients. After 30 minutes of testing, adenosine was given in sinus rhythm and during ventricular pacing; atrioventricular and ventriculoatrial block was demonstrated in all patients during adenosine administration.

### Follow up data

Two patients had early recurrence of AP conduction, noted at 25 and 30 days after the acutely successful catheter ablation. Both patients underwent a second successful cryoablation both utilizing a larger tipped catheter (8mm), again with a His bundle electrogram on the cryoablation catheter. At a median follow-up of 13 months (range: 3 to 37 months) from the last successful catheter ablation, all patients were asymptomatic with no evidence of ventricular preexcitation and a mean PR interval of 155 ± 45 msec on the surface electrocardiogram.

## Discussion

Radiofrequency energy has been used for catheter ablation of accessory pathways in children for over 20 years [8]. The use of radiofrequency energy has proven to be safe and effective in eliminating accessory pathway conduction. However, a septal location of the accessory pathway carries a higher risk of damaging the conduction system during the ablation [3]. Prior reports of ablation of septal accessory pathways using radiofrequency energy have demonstrated a high acute success rate in eliminating accessory pathway conduction (94% - 96%), however, the incidence of heart block was significant (3% to 5%)[2,3,9].On the other hand, other authors [10] reported no atrioventricular block at the expense of a slightly lower acute success rate (88%) for anteroseptal accessory pathways; heart block was avoided by moving the radiofrequency catheter 3 mm away from the largest His deflection.The presence of a His bundle electrogram on the ablation catheter has been shown to be associated with the development of AV block when conducting an ablation to intentionally cause heart block [5]. The electrophysiologist might not be able to predict if a radiofrequency lesion will cause permanent damage of the conduction system, therefore, when mapping the anterior septal area, if a His bundle electrogram is found, it is general practice not to deliver radiofrequency energy in such a location. Since the introduction of transvenous catheter based cryotherapy, multiple studies have shown its efficacy in eliminating accessory pathway conduction without risk of permanent heart block [9,11,12]. Miyazaki et al described the use of cryotherapy for ablation of septal arrhythmia substrates; there were 4 patients in whom a His bundle electrogram was noted on the ablation catheter [13]. However, only one patient had an accessory pathway and there was no preexcitation, the other patients had AV nodal reentry or ventricular tachycardia. In our report, we found that in 8.5% of patients with anteroseptal accessory pathways, the pathway is extremely close to the atrioventricular node and the His bundle, such that on the site of success a His bundle electrogram was recorded on the ablation catheter.

In our retrospective review, we demonstrated that cryotherapy with a His bundle electrogram on the ablation catheter can be done safely without permanent damage of the conduction system. Care should be taken to monitor atrioventricular nodal conduction during the ablation, particularly in patients with ventricular preexcitation in which normal conduction might not be easily seen. Once the catheter freezes and adheres to the endocardial surface, pacing maneuvers can be utilized to monitor atrioventricular nodal conduction. Our techniques included the use of rapid atrial pacing with conduction via the atrioventricular node, atrial extra-stimulus testing with a coupling interval shorter than the effective refractory period of the accessory pathway and ablation during orthodromic reentrant tachycardia. It is imperative that changes in atrioventricular nodal conduction are recognized quickly while the effect of the cryotherapy is still reversible. In terms of the catheter approach to the area, we have used both the right internal jugular vein and the right femoral vein with success from both approaches. Two patients had recurrence of accessory pathway conduction. Similarly, Buddhe et al demonstrated a 33% recurrence rate after ablation of anteroseptal accessory pathways in a long term follow up study [14]. Both patients were successfully ablated during a second procedure using an 8 mm tip cryocatheter without complications. Perhaps consideration should be given to use a larger tip catheter on the first ablation attempt. Our study has the inherited limitations of a retrospective review of a small number of patients.

## Discussion

This report demonstrated that anteroseptal accessory pathways were safely and successfully ablated, in a small number of patients, using cryoenergy even when a His bundle electrogram is recorded from the tip of the ablation catheter.
